# Host populations, challenges, and commercialization of cryptococcal vaccines

**DOI:** 10.1371/journal.ppat.1011115

**Published:** 2023-02-09

**Authors:** Maurizio Del Poeta, Floyd L. Wormley, Xiaorong Lin

**Affiliations:** 1 Department of Microbiology and Immunology, Stony Brook University, Stony Brook, New York, United States of America; 2 Division of Infectious Diseases, Stony Brook University, Stony Brook, New York, United States of America; 3 Institute of Chemical Biology and Drug Discovery, Stony Brook University, Stony Brook, New York, United States of America; 4 Veteran Administration Medical Center, Northport, New York, United States of America; 5 Department of Biology, Texas Christian University, Fort Worth, Texas, United States of America; 6 Department of Microbiology, University of Georgia, Athens, Georgia, United States of America; University of Maryland, Baltimore, UNITED STATES

## Abstract

Vaccines are one of the most effective public health tools to prevent and manage infectious diseases. Since the first clinical use of vaccines in the late 18th century, many vaccines have been successfully developed to combat bacterial and viral infections, including the most recent Coronavirus Disease 2019 (COVID-19) pandemic. However, there remains no vaccine that is clinically available to treat or prevent invasive fungal diseases, including cryptococcal meningoencephalitis. This fungal disease is uniformly fatal without treatment and has a global mortality rate of over 70%. Despite a dire need for an effective cryptococcal vaccine, there are many scientific and economic challenges to overcome prior to making it a reality. Here, we discuss some of these challenges as well as steps that the community is taking for commercialization of effective cryptococcal vaccines.

## Main text (Q/A format)

### 1. What are the targeted host populations for cryptococcal vaccines?

The pathogenic cryptococcal species is composed of the *Cryptococcus neoformans* species complex and the *Cryptococcus gattii* species complex. *C*. *neoformans* is considered an opportunistic pathogen and is distributed worldwide. This fungus is commonly isolated from avian excreta, soil, and trees. *C*. *neoformans* is responsible for 99% of cryptococcosis cases and has been recently listed as one of the 4 critical fungal pathogens by the World Health Organization (WHO) [1]. *C*. *gattii* is considered as a primary pathogen and is associated with trees. Compared to *C*. *neoformans*, *C*. *gattii* is more commonly isolated in tropical or subtropical regions, but its geographical range has clearly expanded to include more temperate climates such as the Pacific Northwest of the United States and Mediterranean Europe [2,3]. Epidemiology studies suggest that people are exposed to infectious propagules of these pathogenic cryptococcal species in early childhood, and such exposures are often asymptomatic because the fungus is either cleared or becomes dormant in the lungs [4–6]. However, in people with impaired immunity, dormant fungal cells can reactivate, disseminate through the blood stream, and cross the brain–blood barrier. Systemic cryptococcosis, most commonly manifested clinically as cryptococcal meningoencephalitis, is fatal without treatment. Even with the current antifungal therapy, the global mortality rates are about 70% because of late diagnosis, limited options of antifungals, unsatisfactory drug efficacy, and drug toxicity issues. Cryptococcal meningoencephalitis is responsible for 15% of AIDS-related deaths globally and is the third leading fungal infection in solid organ transplant patients [7,8]. It is commonly accepted that systemic cryptococcosis is often caused by reactivation of a dormant infection that could have been decades old, although acute infections after exposure to a large dose of this fungus could also occur. In addition to HIV^+^ patients who develop AIDS, other high-risk populations include those undergoing corticosteroid therapy or experiencing chronic renal or hepatic failure, rheumatologic disorders, chronic lung disease, and malignancy. There are also increased incidences of cryptococcosis in individuals without apparent immuno-compromising conditions [9–12].

Thus, we believe that the host population to be targeted for cryptococcal vaccination should be primarily individuals predicted to have an exceptionally high risk for developing cryptococcosis (i.e., HIV^+^ individuals or patients prior to receiving solid organ transplants or starting immunosuppressing therapies) and some immune-competent persons in *C*. *gattii* endemic areas.

### 2. What are the scientific challenges of developing effective cryptococcal vaccines?

There are several major scientific challenges in developing effective cryptococcal vaccines. (**1**) Most vaccines are prophylactic and work by preventing or mitigating severity of potential future infections. However, due to likely universal exposure to this environmental fungus at a young age and potential general asymptomatic colonization, the ideal cryptococcal vaccines should be both preventative and therapeutic. As mentioned earlier, many systemic cryptococcosis cases result from reactivation of previously asymptomatic infections upon host immunosuppression due to HIV infection or following receipt of immunosupressing medications. (**2**) Many vaccines work in immunocompetent individuals but they are not as effective in immunocompromised patient populations. Unfortunately, immunocompromised individuals have the highest risk of developing systemic cryptococcosis, particularly persons with suppressed CD4^+^ T cell-mediated immune responses. Thus, for a vaccine to be meaningful in combating cryptococcosis, the vaccine would need to be protective in immunocompromised individuals. This presents a challenge as most vaccines rely on the collaboration of innate and adaptive immunity for their efficacy. That said, preclinical studies in animal models indicate that some anti-cryptococcal vaccines could be effective in immunocompromised patients [13–15]. (**3**) *C*. *neoformans* and *C*. *gattii* diverged from a common ancestor approximately 50 million years ago [16]. Even within the *C*. *neoformans* species complex, serotype A diverged from serotype D about 25 million years ago. In comparison, humans split from rodents about 90 million years ago. Thus, the relatively long evolutionary distance between different cryptococcal pathogenic isolates with the associated genetic and phenotypical diversity presents a challenge to develop a vaccine that will work for isolates from all lineages. Fortuitously, *C*. *neoformans* serotype A strains cause the vast majority of the disease, including >99% of infections in AIDS patients and >95% of overall cryptococcal cases. Thus, a vaccine effective against only serotype A isolates will make a significant difference in the overall fight against cryptococcosis. (**4**) Unlike viruses, *Cryptococcus* is a free-living eukaryotic organism with complex biology and life cycle. The genome of *Cryptococcus* carries about 7,000 protein-coding genes and more than 1,000 long noncoding RNA genes. It is difficult to predict which genes encode potential immunogens. Experimental evidence indicates that some cytosolic proteins are somehow secreted (e.g., Gpd1) and could be immunogenic [17]. To further compound the issue, this is the only pathogenic fungus that is wrapped by a capsule, which is composed of mostly polysaccharides with proteins/glycoproteins being a minor component. Cryptococcal capsule size varies widely depending on the culture conditions and is continuously shed [18,19]. The effect of capsule on vaccination is also likely to be complicated. The polysaccharide capsule masks the highly antigenic cell wall while also serving as a site where potentially immunogenic and protective factors accumulate. Acapsular mutants, while being easily recognized by the host due to the exposed cell wall and incapable of causing disease, have the potential to elicit strong proinflammatory responses but are not usually very effective as vaccines [20–23]. For example, immunoprotection elicited by the live sterylglucosidase *sgl1*Δ mutant, which accumulates sterylglucosides, requires capsule [21]. Likewise, immunoprotection elicited by heat-killed morphogenesis *ZNF2*^oe^ strain, which displays increased antigens on the cell exterior, requires capsule [22]. The protein subunit vaccines (Cda2, MP88, etc.) work more effectively when encapsulated in glucan particles [24,25]. It is not clear if either a polysaccharide cell wall component or the polysaccharide capsule is required to induce robust immunity against cryptococcosis.

These factors need to be considered when developing vaccines against cryptococcal meningitis. Three classes of vaccines that could potentially work include: whole organism vaccines, subunit recombinant protein vaccines, and mRNA vaccines. Live attenuated strains are most effective at inducing long-lasting protective immune responses. Such vaccines are the oldest and have been used to elicit protective immunity against viral and bacterial disease (e.g., tuberculosis). However, live attenuated strains are unlikely to be adopted for cryptococcal vaccines given that immunocompromised individuals are at high risk of developing cryptococcosis and the risk for vaccination with live attenuated strains to result in disease is too high. Inactivated whole organism vaccines provide a safer option. Multiple inactivated cryptococcal mutant strains and even inactivated wild-type cells have been demonstrated to induce highly effective and long-lasting immunity against cryptococcosis in animal models [26–28]. However, higher inocula of inactivated whole cells are needed to induce host immunity comparing to levels of immunity observed using live attenuated strains. Furthermore, given the complexity of the fungal cell surface, it is challenging—if not impossible—to predict potential complications if such vaccines are given to humans (e.g., cross-reactivity between fungal and host molecules as in the case of the autoimmune paralysis observed during Guillain–Barré syndrome due to cross-reaction between some bacterial lipooligosaccharides and human glycolipids like GM1 gangliosides). Unfortunately, these types of complications oftentimes only become evident during human clinical trials. To circumvent this issue, subunit recombinant protein vaccines administered with an adjuvant provide a safer class of vaccines. Preclinical studies indicate that encapsulating recombinant proteins within glucan particles is potentially a more effective vaccine delivery option compared to coupling the recombinant proteins with conventional adjuvants. However, it is unknown if glucan particles could have adverse effects in humans, given the universal exposure to fungi/yeasts that carry β-glucans (skin, respiratory and digestive tracks).

A new but clinically proven vaccine technology is mRNA vaccines. However, delivering stabilized mRNAs encoding immunogens packaged in lipid nanoparticles (LNPs) has not been developed for any fungal disease. Expressing mRNAs that encode known protective immunogens in the host cell could potentially work better than recombinant protein vaccines because these immunogens will be glycated, and hence, more closely resemble proteins produced by the fungus *C*. *neoformans* in contrast to recombinant proteins produced by *Escherichia coli* that are commonly used in subunit vaccines. Furthermore, it is relatively simple to include mRNAs encoding multiple different immunogens in LNPs, which could further boost host immunity. Combinatorial vaccines (either recombinant protein vaccines or mRNA vaccines) might be necessary to elicit robust host responses because fungal pathogens are far more genetically and biochemically complex than viruses. One potential issue with subunit protein vaccines and mRNA vaccines, in comparison to whole-cell vaccines, is waning immunity. Short duration of protection will create practical difficulties in vaccinating the high-risk populations. The production cost of mRNA vaccines and subunit recombinant protein vaccines are likely much higher than whole organism vaccines.

### 3. Are there any benefits to couple cryptococcal vaccines with antifungal therapy?

It is ideal but unlikely that vaccines will be 100% protective to prevent mortality and symptomatic diseases in all individuals, particularly those who are immunodeficient. Thus, coupling prophylaxic antifungal therapy with vaccination could prevent potential activation of cryptococcal infection in high-risk individuals. Coupling vaccination with aggressive antifungal therapy in patients with active invasive infections could potentially shorten the duration of the therapy and increase the success rates. However, such benefits would have to be first demonstrated in preclinical studies since these studies have not yet been explored experimentally.

Antifungal therapy for cryptococcal meningitis is typically composed of 3 phases: induction therapy, consolidation therapy, and then maintenance therapy. The recommended induction therapy based on recent WHO guideline includes a variation of amphotericin B (with liposome formulation preferred), flucytosine and fluconazole combinations depending on the availability of the drugs (AmB and flucytosine are not always available in resource-limited areas). Consolidation and maintenance therapy typically rely on fluconazole, with a higher dose for the consolidation phase and a lower dose for the maintenance phase of the therapy. However, given that relapses from patients treated with antifungal therapy are frequent, and about 90% of relapses are caused by the original strains [29], antifungal therapy alone does not always result in fungal clearance. The cost of current antifungal therapy is also high. For a single individual with cryptococcosis, hospital in-patient medical costs in the United States range from $40,000 to $70,000 [9–12,30,31]. Thus, coupling vaccines with antifungal therapy could help eradicate the fungus and prevent recurrent infections. Moreover, such coupling could potentially reduce the duration of antifungal therapy, thereby also reducing drug toxicity to the patient and the cost associated with the treatment.

### 4. What are the critical steps towards commercialization?

In general, commercialization of a product—a vaccine or a drug—requires a clear understanding of the critical steps necessary to evaluate whether the product is worth manufacturing for a particular market. This is necessary because commercialization relies heavily on the concept that the business should be viable (i.e., profitable).

One critical step is the understating of the costs associated with the introduction of a new vaccine. The Department of Vaccine and Biologicals at WHO provides guidelines for estimating costs of introducing new vaccines [32]. The vaccine costs per year, c, are estimated as c = p × n, where p is the price per dose of the new vaccine and n is the number of doses administered per person. The price per dose (p) is dictated mainly by the cost for manufacturing the dose and it should be kept <$5/dose, better if approximately $2/dose, if the vaccine is mainly for a low-income country (https://www.unicef.org/supply/pricing-data). The cost for the number of doses is much more complex because it includes a series of variables, such as the rate of immunization coverage (i), the cohort(s) (b), the number of doses per fully immunized person (d), the wastage rate (w), and the reserve stock (r). Thus, the formula becomes: c = p × {i × b × d × [1/(1—w)] × (1+r)}. If we consider only the HIV^+^ population in the sub-Saharan Africa (approximately 20 million) [33] and we aim to immunize all subjects with advanced HIV disease (AHD) (approximately 30%) [34–36], which is the cohort mostly at risk of developing cryptococcosis, the number of subjects in the sub-Saharan Africa eligible for a cryptococcal vaccine would be approximately 6.5 million. This is assuming that all people living with HIV in sub-Saharan Africa know their status, which is not the case. But, if anything, this number of individuals will most likely be an underestimate.

Based on the formula above, the cost of a cryptococcal vaccine will be approximately $48,750,000, assuming a price per dose of $2 (p), 70% immunization coverage (i), a 6.5 M cohort (b), 3 doses per fully immunized person (d), a 30% waste (r), and a 25% dose in stock after 1 year. This cost will only apply to the first year of vaccination and does not consider the cost associated with the vaccination of new AHD cases in subsequent years after the first year and/or if there will be a need for subsequent booster vaccinations.

Additional costs will include the logistics in delivering and distributing the vaccine, such as transportation costs, storage, and the personnel involved in managing the new vaccine (e.g., coordinator/manager, surveillance staff, community health workers, nurses, and doctors). Formula to estimate these costs are available but they may vary significantly depending on whether the new vaccine can use already existing infrastructures that distribute current vaccines or whether a total new distribution channel needs to be created. Finally, other costs related to the introduction of a new vaccine are social mobilization, training of healthcare staff, new immunization forms, and stationery.

The People Vaccine Alliance (PVA) reported that Pfizer, BioNTech, and Moderna are making combined $65,000/minute for selling their COVID-19 vaccine [37]. They have sold the majority of doses to resource-rich countries. In fact, according to PVA, Pfizer sold only 1% and Moderna sold just 0.2% of their vaccine to low-income countries. And this is for COVID-19, a disease afflicting almost every single person on the planet, thus requiring vaccination of the entire world population. So, how can we envision a financially viable cryptococcal vaccine that will be delivered to a relatively small number of individuals (approximately 6.5 M), such as those with AHD, mostly living in low-income countries? Clearly, under the slogan “profit being necessary for commercialization,” a vaccine against cryptococcosis will not be feasible, because it will be difficult, if not impossible, to engage a pharmaceutical company willing to manufacture it for approximately $2/dose.

But we argue that framing the discussion on whether a cryptococcal vaccine should be “for profit” or “not for profit” may be deceiving. In fact, for pharmaceutical companies, “for profit” almost always means “for *high/maximal* almost *obscene* revenue” instead of generating a gain that exceeds the expenses, the costs, and taxes involved in sustaining the activity of manufacturing the vaccine. Alternatively, small and local pharmaceutical companies in the countries where the cryptococcal vaccine is most needed can be trained to manufacture the vaccine. Several low-income countries have public sectors for vaccine manufacturing. These sectors may not be equipped to produce COVID-19 vaccines at the large scale required to supply the entire population of the country [38], but it may be feasible for them to produce a cryptococcal vaccine directed to a relatively small population.

Importantly, the Global Alliance for Vaccines and Immunization (GAVI) and other organizations are committed to support vaccine manufacturing in Africa, by creating “regional hubs” to produce vaccine(s) that will be sold in low-income countries. The scientific community, governments, and the World Trade Organization are moving forward to smooth the rules and regulations with sharing intellectual property, particularly in case of emergency use [39,40].

For local governments of low-income countries, it is time to shift away from the reasoning that cost for a new vaccine is just another expenditure to their limited budget. Instead, these governments should consider the savings that the vaccination strategy will bring on the health care system by preventing the costs associated with treatment or/and hospitalization of the subjects affected by that disease. For example, even if we consider a mere 3% prevalence of cryptococcosis among ADH patients (approximately 195,000 subjects—a very conservative figure) [36,41,42] and a treatment cost of approximately $2,000 per patient for only 2 weeks of the induction therapy with amphotericin B and flucytosine [43], the total cost of treatment is $390 M. This cost is approximately 8-fold the cost necessary to vaccinate the entire ADH population of sub-Saharan Africa.

This is without considering the healthcare costs associated with hospitalization, although this will vary significantly from country to country and from hospitals to hospitals. On average, in South Africa, the average length of stay for a patient with the first episode of cryptococcosis is 17 days [43,44] with an estimate average baseline of stay of approximately 41.5 days/year. Considering an average hospitalization cost of approximately $145/day, the hospitalization cost in South Africa will be $6,017/patient/year. In Uganda, hospitalization costs are much lower, estimated at $40 per patient/day for a total cost of $1,640/patient/year affected with cryptococcosis. If we take an average of approximately $3,800/patient/year, we can estimate a total cost of approximately $248 M in hospitalization cost if only approximately 30% (approximately 65,000) of patients at risk of developing cryptococcosis will require hospitalization. If we now combine the cost of treatment and the cost of hospitalization, we are reaching approximately $638 M/year.

In United States, there are about 1 M people living with HIV and approximately 20% (200,000) of these subjects have ADH based on CDC reports. Based on the formula above c = p × n, it would cost merely approximately 1.5 M to vaccinate the entire ADH population in United States. The economic burden of cryptococcal meningitis in United States has been estimated at approximately $38,000/patient/year for hospital costs (including treatment), reaching a cumulative total cost of approximately $100,000/person over 5 years [45–47]. So, if we assume an average cost of $20,000/person/year and a prevalence of cryptococcal meningitis of only 1% (approximately 2,000 subjects) among the ADH patients in United States, which is below the estimates [48,49], the total healthcare costs for these patients will be approximately $40 M/year, which is approximately 27-fold more than the cost needed to vaccinate the entire ADH population in Unites States.

Obviously, a vaccine will not protect 100% of the subjects at risk, but even if the vaccine only protects 50% to 70% of the ADH population, the overall savings that the vaccination strategy will bring over a 5-year period are simply enormous.

Thus, from a government point of view, profitability should be considered by investing in strategies in preventing diseases that will significantly and effectively reduce healthcare costs and drug expenditure and improve the health of their citizens while also supporting the local economy. Governments could argue that by implementing such preventive strategies, they will face pressure from pharmaceutical industries, as their drug sale for the treatment of those diseases would drastically, and inevitably, decrease. This is a possibility but if we learned anything from COVID-19 pandemic is that vaccination not only aims to prevent the infection but also, importantly, to decrease the symptomatology and the severity of the disease. So, drug companies should not fear to suddenly throw their product out of the window.

### 5. What the research community need to do to achieve commercialization of cryptococcal vaccines?

Few cryptococcal vaccines have been proposed by the research community. These vaccines, live-attenuated or killed, have been showed to be highly protective in various mouse models of cryptococcal meningitis (reviewed in [50]). It is now time for the research community to come together to complete the critical steps needed for the preclinical phase, to file an Investigational New Drug (IND) application with the Food and Drug Administration (FDA), and to move the testing in human subjects. A discussion with the Center for Biologics Evaluation and Research (CBER) at the FDA should be initiated during the preclinical phase, as they can guide through the process of collecting key data necessary for the IND portfolio.

One way to do this is to move forward with each vaccine in parallel. However, we argue that this strategy may compete for the same resources and slow down the entire preclinical phase. A better way is to consolidate and combine the effort through a Product Development Partnerships (PDP). Although there are pros and cons about the PDP strategy [51–53], here is a proposal on how this can be achieved (S1 Fig).

1. Call for a meeting among the interested community and define the lead person(s) or a program manager. The person(s)/manager should be familiar with the milestones of the preclinical phase. This person will be the overall coordinator, should have financial and legal support through a start-up or a larger company/organization, and will be an intermediate among various principal investigators working on cryptococcal vaccines as well as with the CBER at the FDA. This management could also be done by a new or an existing PDP organization, such as Lygature [51].
2. Whereas a start-up or a collaboration with an existing company or a PDP organization will be initiated, it is essential to finalize who holds the intellectual property (IP) of the vaccine(s). This will require a *license agreement* for the background Intellectual Properties (IP), a *collaborative agreement* among all individuals involved and a *management plan* between the company/organization and the universities involved, so that ideas and data can be freely shared and used by the company/organization to move the product forward. This is not trivial, particularly from the university perspective, as these universities will all be competing for technical and financial resources.
3. Work on the actual product(s), such as which vaccine to select, which formulation to consider, and who is doing what. This will require new funding because existing funding may relate to basic science studies and not to the research and development of a product. Besides, it will be difficult, if not impossible, for the company to oversight the spending of fundings assigned to universities. So, although each investigator/university may perform different and complementing tasks, the funding for these tasks should go to the company/organization, which will then subcontract each investigator/university for specific tasks. When the product is more advanced, outside companies can be subcontracted for additional tasks related specifically to the final development of the product (e.g., scale-up production, final formulation, large scale toxicology, and large-scale PK studies).
4. Submit the IND to the FDA. Funding for this should come from the company/organization or through a partnership with a large company. This work will be facilitated by engaging with the SBER early to determine the critical, essential data needed for the portfolio. This will maximize and prioritize the tasks and milestones in step 3.
5. Acquire necessary funding to perform clinical trials in human subjects. The cryptococcal community will greatly benefit from having already existing infrastructures and collaborations with various centers and hospitals in Africa that are involved in testing new diagnostics and new drug formulations in patients with cryptococcosis.
6. Bring the product into the market and make it available to the entire ADH population. As discussed above, this can be done in partnership with local companies (e.g., Afrigen in South Africa), which already make vaccines for low-income countries.

In 2021, a strategic framework from South Africa and from the Global Action for Fungal Infections (GAFFI) is aiming to end cryptococcal meningitis death by 2030 [54]. This seems to be an ambitious goal but the strategic framework outlines clearly the diagnostic needs and the treatment needs that should significantly improve the management of existing patients. Addressing these needs, such as more affordable point-of-care diagnostics and increased availability and better formulations of current treatments, will definitively improve the outcome of cryptococcal meningitis in the coming years. But we also need to encourage and improve the research and development of new therapeutics and new preventive strategies, such as vaccines. Although these preventive approaches are challenging, time-consuming, and require significant effort, the community is already united and ready to start implementing these important strategies. The WHO recently listed *Cryptococcus neoformans* in the *critical* group of 4 fungi for which a major effort from governments and researchers is needed for an effective response [1]. The time to act is upon us.

## Supporting information

S1 Fig Flow chart of the steps towards a cryptococcal vaccine. (TIF)
