# Supplementary figures and images for: Host populations, challenges, and commercialization of cryptococcal vaccines
Source: PLoS Pathog. 2023 Feb 9;19(2):e1011115. doi: 10.1371/journal.ppat.1011115 (PMC9910758; doi:10.1371/journal.ppat.1011115)

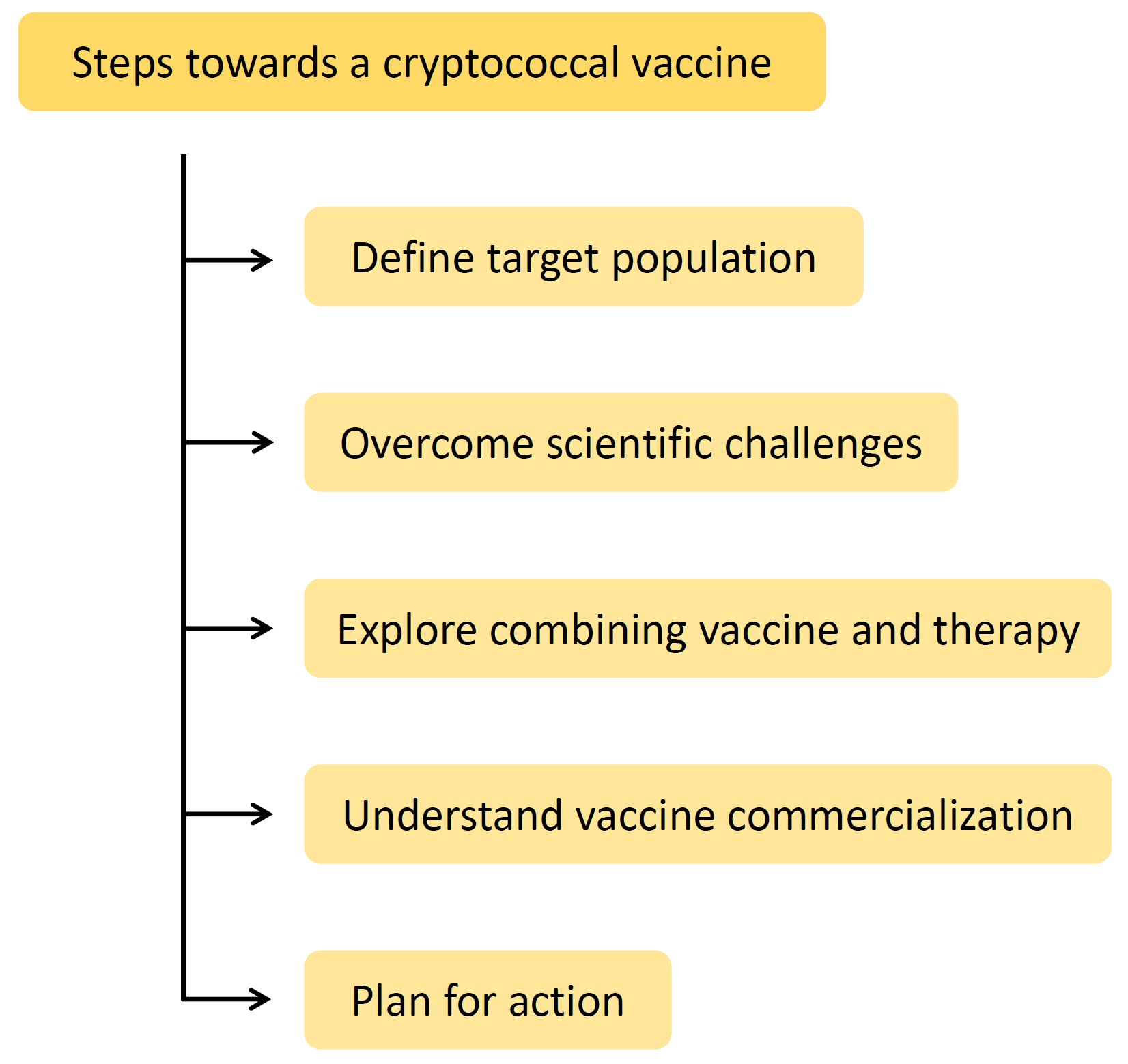

Supplement: S1 Fig — (TIF) [file ppat.1011115.s001.tif]
